# High-temporal resolution DCE-MRI improves assessment of intra- and peri-breast lesions categorized as BI-RADS 4

**DOI:** 10.1186/s12880-023-01015-4

**Published:** 2023-04-19

**Authors:** Yufeng Liu, Shiwei Wang, Jingjing Qu, Rui Tang, Chundan Wang, Fengchun Xiao, Peipei Pang, Zhichao Sun, Maosheng Xu, Jiaying Li

**Affiliations:** 1 Department of Radiology, The First Affiliated Hospital of Zhejiang Chinese Medical University (Zhejiang Provincial Hospital of Chinese Medicine), 54 Youdian Road, Hangzhou, China; 2 The First Clinical Medical College of Zhejiang Chinese Medical University, Hangzhou, China; 3 Department of Radiology, The Second Affiliated Hospital of Zhejiang University School of Medicine, Hangzhou, China; 4 Department of Pathology, The First Affiliated Hospital of Zhejiang Chinese Medical University (Zhejiang Provincial Hospital of Chinese Medicine), Hangzhou, China; 5 GE Healthcare, Precision Health Institution, Hangzhou, China

**Keywords:** Breast cancer, Magnetic resonance imaging, Dynamic contrast-enhanced MRI, Breast Imaging Reporting and Data System category 4

## Abstract

**Background:**

BI-RADS 4 breast lesions are suspicious for malignancy with a range from 2 to 95%, indicating that numerous benign lesions are unnecessarily biopsied. Thus, we aimed to investigate whether high-temporal-resolution dynamic contrast-enhanced MRI (H_DCE-MRI) would be superior to conventional low-temporal-resolution DCE-MRI (L_DCE-MRI) in the diagnosis of BI-RADS 4 breast lesions.

**Methods:**

This single-center study was approved by the IRB. From April 2015 to June 2017, patients with breast lesions were prospectively included and randomly assigned to undergo either H_DCE-MRI, including 27 phases, or L_DCE-MRI, including 7 phases. Patients with BI-RADS 4 lesions were diagnosed by the senior radiologist in this study. Using a two-compartment extended Tofts model and a three-dimensional volume of interest, several pharmacokinetic parameters reflecting hemodynamics, including K^trans^, K_ep_, V_e_, and V_p_, were obtained from the intralesional, perilesional and background parenchymal enhancement areas, which were labeled the *Lesion*, *Peri* and *BPE* areas, respectively. Models were developed based on hemodynamic parameters, and the performance of these models in discriminating between benign and malignant lesions was evaluated by receiver operating characteristic (ROC) curve analysis.

**Results:**

A total of 140 patients were included in the study and underwent H_DCE-MRI (n = 62) or L_DCE-MRI (n = 78) scans; 56 of these 140 patients had BI-RADS 4 lesions. Some pharmacokinetic parameters from H_DCE-MRI (Lesion_K^trans^, K_ep_, and V_p;_ Peri_K^trans^, K_ep_, and V_p_) and from L_DCE-MRI (Lesion_K_ep_, Peri_V_p_, BPE_K^trans^ and BPE_V_p_) were significantly different between benign and malignant breast lesions (*P* < 0.01). ROC analysis showed that Lesion_K^trans^ (AUC = 0.866), Lesion_K_ep_ (AUC = 0.929), Lesion_V_p_ (AUC = 0.872), Peri_K^trans^ (AUC = 0.733), Peri_K_ep_ (AUC = 0.810), and Peri_V_p_ (AUC = 0.857) in the H_DCE-MRI group had good discrimination performance. Parameters from the BPE area showed no differentiating ability in the H_DCE-MRI group. Lesion**_**K_ep_ (AUC = 0.767), Peri_V_p_ (AUC = 0.726), and BPE_K^trans^ and BPE_V_p_ (AUC = 0.687 and 0.707) could differentiate between benign and malignant breast lesions in the L_DCE-MRI group. The models were compared with the senior radiologist’s assessment for the identification of BI-RADS 4 breast lesions. The AUC, sensitivity and specificity of Lesion_K_ep_ (0.963, 100.0%, and 88.9%, respectively) in the H_DCE-MRI group were significantly higher than those of the same parameter in the L_DCE-MRI group (0.663, 69.6% and 75.0%, respectively) for the assessment of BI-RADS 4 breast lesions. The DeLong test was conducted, and there was a significant difference only between Lesion_K_ep_ in the H_DCE-MRI group and the senior radiologist (*P* = 0.04).

**Conclusions:**

Pharmacokinetic parameters (K^trans^, K_ep_ and V_p_) from the intralesional and perilesional regions on high-temporal-resolution DCE-MRI, especially the intralesional K_ep_ parameter, can improve the assessment of benign and malignant BI-RADS 4 breast lesions to avoid unnecessary biopsy.

## Background

Breast magnetic resonance imaging (MRI) plays a pivotal role in the screening and diagnosis of breast tumors due to its noninvasive and highly sensitive nature [1]. MRI can detect lesions in women with dense breast glandular tissue, compensating for the shortcomings of mammography (radioactivity, easily missing diagnoses, etc.), and can help address equivocal findings on mammography and ultrasound [1–3]. Conventional dynamic contrast-enhanced MRI (cDCE-MRI) of the breast mainly provides qualitative characteristics of breast disease, such as the time–signal intensity curve (TIC) and morphological type. cDCE-MRI has high spatial resolution and variable specificity values ranging from 47 to 97% for the identification of focal breast lesions [4].

In clinical practice, the Breast Imaging Reporting and Data System (BI-RADS) MRI lexicon is widely used, and diagnoses are made according to a combined analysis of lesion morphology and the TIC curve by radiologists [5]. Among BI-RADS assessment categories, BI-RADS category 4 indicates an abnormality that is suspicious for malignancy with a range from 2 to 95% [6]. The invasive biopsy is recommended for BI-RADS 4 lesions, implying that numerous benign lesions are unnecessarily biopsied [7]. Although the overall sensitivity of cDCE-MRI for breast cancer is very high, some malignant lesions can be missed because there is some overlap between the features of atypical malignant lesions and benign lesions [8]. It is necessary to find an objective biomarker to improve the assessment of BI-RADS 4 lesions and to correctly recognize benign and malignant lesions.

Benign and malignant breast lesions have different characteristics in terms of angiogenesis and vascular permeability [9]. Quantitative analysis of DCE-MRI can objectively provide multiple pharmacokinetic parameters that can reflect tumor vascularity and permeability [10]. These parameters show diagnostically useful changes earlier than the morphologic parameters. However, breast cDCE-MRI typically lasts more than 60 s, which may obscure important kinetic information in the early phase. High-temporal-resolution DCE-MRI (hDCE-MRI) of the breast can be performed in a few seconds to better evaluate the characteristics of tumor microvessels. Khouli et al. [11] proposed that quantitative pharmacokinetic parameters from hDCE-MRI were similar to conventional kinetic curve analysis in the degree to which they improved in diagnostic performance. Thus, it is necessary to further compare the performance of pharmacokinetic parameters obtained from high- and low-temporal resolution DCE-MRI, especially in patients assessed as having BI-RADS 4 lesions.

In recent years, background parenchymal enhancement (BPE) has attracted a great deal of interest and has been added to the BI-RADS MRI lexicon [5]. BPE can be described as the enhancement of normal breast tissue on breast MRI after intravenous administration of a contrast agent [12]. The phenomenon of BPE may represent breast metabolic activity, which is related to tissue vascularity and permeability [13]. Most studies have found that higher levels of BPE were associated with a higher risk of breast cancer and subsequently higher rates of biopsy recommendations [12–14]. Therefore, in this study, we aimed to investigate whether high-temporal-resolution DCE-MRI would be superior to conventional DCE-MRI for differentiating between malignant and benign BI-RADS 4 breast lesions using pharmacokinetic parameters from the intralesional, perilesional and background parenchymal enhancement areas.

## Methods

### Patients

**Fig. 1:**
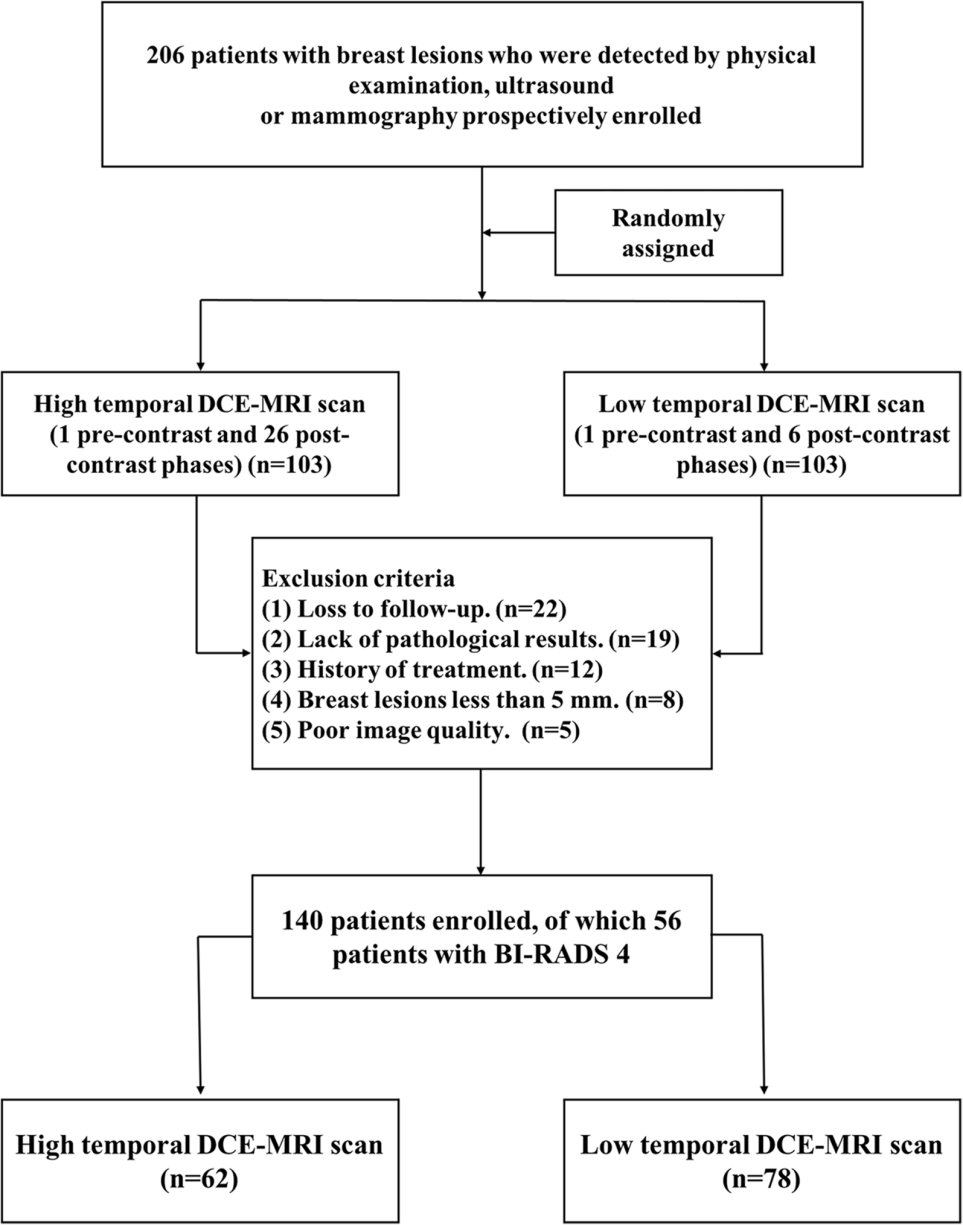
The flowchart of patient selection

The study was approved by the institutional review board, and written informed consent was obtained. From April 2015 to June 2017, patients at our center who had suspicious breast lesions were prospectively included in the study and randomly assigned to undergo either high-temporal-resolution (27 phases) or conventional low-temporal-resolution (7 phases) DCE-MRI. MRI was performed in the second week of the menstrual cycle in premenopausal patients. The inclusion criteria included the following: (1) breast lesions were found through either physical examination or imaging, such as mammography or ultrasound; (2) no surgery, biopsy or medication for treating breast lesions was conducted or administered before the DCE-MRI scan; and (3) all patients were ≥ 18 years old. The exclusion criteria were as follows: (1) loss to follow-up or lack of pathological results; (2) breast lesions less than 5 mm; (3) any prior treatment of the lesions; and (4) poor image quality. Finally, a total of 140 patients met the above criteria, of which 56 patients had BI-RADS 4 in this study. The flowchart of patient selection is shown in Fig. 1.

### MRI protocol

DCE-MRI examination was performed on a 3.0T MR scanner (Magnetom Verio, Siemens Medical Solutions, Erlangen, Germany) with a 16-channel phased-array breast coil. The patient was placed in the prone position with both breasts naturally suspended in the double coil, and their head was positioned toward the machine. The MRI examination protocols were as follows: 3D positioning scan, cross-sectional turbo inversion recovery magnitude (TIRM) sequence (TR = 4000 ms, TE = 70 ms, slice thickness = 4 mm, FOV = 34 cm × 34 cm, matrix = 448 × 448, NEX = 2); bilateral sagittal T2-weighted imaging (T2WI, fat suppression) with the following parameters: TR = 4650 ms, TE = 85 ms, slice thickness = 4 mm, layer space = 1.0 mm, FOV = 20 cm × 20 cm, matrix = 320 × 224, NEX = 4.

3D T1WI Dyna View sequences using low-temporal-resolution DCE-MRI scans were conducted with the following parameters: TR = 4.51 ms, TE = 1.61 ms, slice thickness = 1 mm, FOV = 34 cm × 34 cm, matrix = 448 × 448, and NEX = 1. The sequence had a temporal duration of 60 s and a total of 7 phases (1 pre-contrast and 6 post-contrast phases). The overall scan time was 7 min 9 s. The high-temporal-resolution DCE-MRI scans were obtained using the following parameters: TR = 4.43 ms, TE = 1.38 ms, slice thickness = 2 mm, FOV = 30 cm × 30 cm, matrix = 224 × 224, NEX = 1, and flip angle = 15°. The temporal duration was 11 s, and there were 27 phases (1 pre-contrast and 26 post-contrast phases) in total. The overall scan time was 5 min 3 s.

The contrast agent gadopentetate dimeglumine (Omniscan, GE Healthcare) at a dose of 0.1 mmol/kg was administered by intravenous bolus injection using a double-cylinder high-pressure injector (MR injection system) at a flow rate of 2.0 ml/sec, followed by a 10 ml saline flush.

### Image postprocessing and analysis

**Fig. 2:**
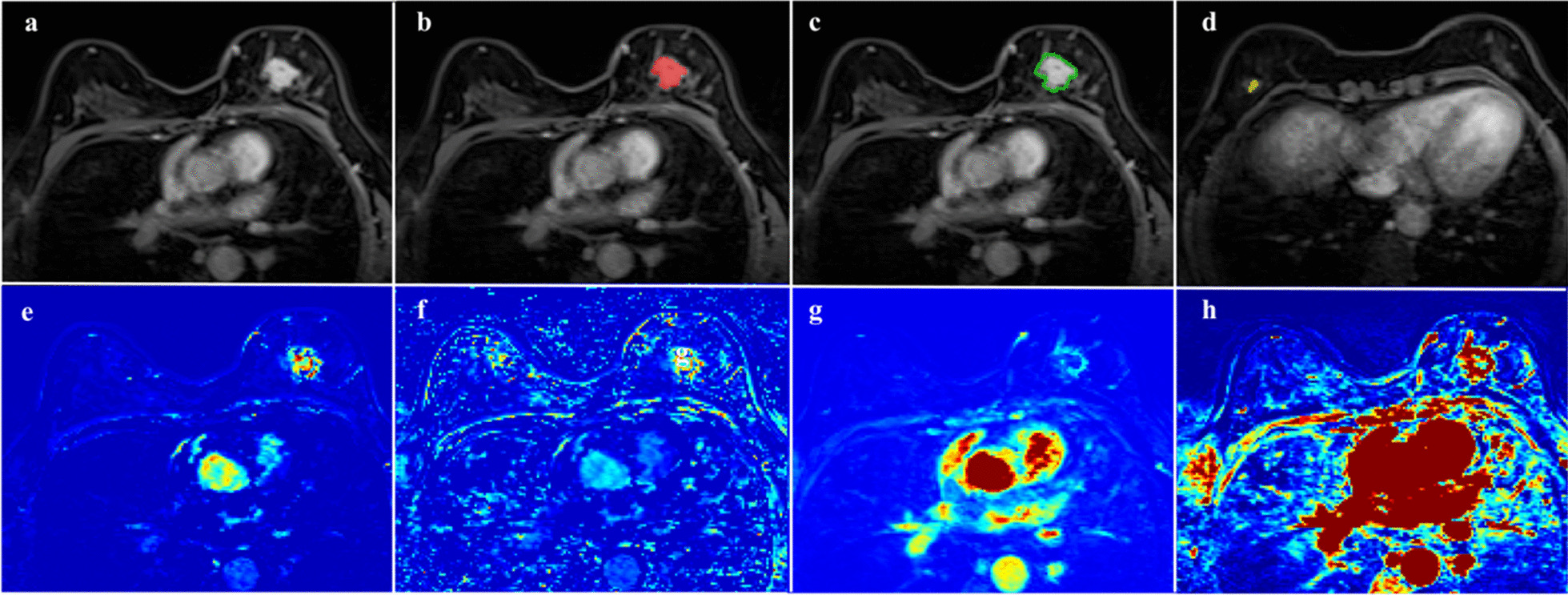
A 51-year-old female with BI-RADS 4c. A mass was clearly identified in the left breast on enhanced T1-weighted imaging (**a**–**d**), K_ep_ (**e**), V_p_ (**f**), K^trans^ (**g**), and K^trans^ map (**h**) from high-temporal-resolution DCE-MRI. All ROIs were manually drawn to cover the intralesional area (**b**, red color), perilesional area (**c**, green color), and background parenchymal enhancement area (**d**, yellow color). The pathological result was breast invasive ductal carcinoma (grade III)

All DCE-MRI scans were transferred into Omni-Kinetic software (GE Healthcare, version 2.10) to obtain the K^trans^ texture features and the pharmacokinetic parameters (K^trans^, K_ep_, V_e_ and V_p_) of the intralesional area (Lesion), perilesional area (Peri) and background parenchyma enhancement area (BPE) in the malignant and benign groups. The specific arterial input function (AIF) was drawn on the thoracic aorta. A two-compartment extended Tofts model and a three-dimensional volume of interest (3D-VOI) were used to obtain perfusion parameters. The specific calculations for the extended Tofts model have been described in detail previously [11]. The region of interest (ROI) was manually selected and delineated on each slice to cover the intralesional, perilesional and BPE areas (Fig. 2). When patients had multiple or bilateral lesions, the largest lesion was selected. The necrotic area and blood vessels inside the lesion were excluded. The perilesional area (Peri) was the region by dilating the lesion border by 2.5–5.0 mm, depending on pixel size [15], and the BPE area was defined by background parenchyma enhancement of normal breast tissue. The ROI for BPE was placed in the area with the strongest background parenchyma enhancement in the contralateral breast and delineated in all slices along the edge of the enhancement area. Each ROI was first identified and delineated by a breast radiologist with 8 years of experience, then verified by another breast radiologist with 15 years of experience**.** Both of these radiologists were blinded to the pathological results.

Pharmacokinetic parameters, including K^trans^ (ml/min; volume transfer constant, defined as the rate of blood leakage to the extravascular extracellular space (EES)), K_ep_ = K^trans^/V_e_ (ml/min; reverse reflux rate constant, defined as the rate of blood leakage from the EES back to blood vessels), V_e_ (fractional EES volume, defined as the proportion of EES volume out of the total volume occupied by the contrast agent), and V_p_ (fractional plasma volume, defined as the proportion of plasma volume out of the total volume occupied by the contrast agent), were used to quantitatively evaluate the microcirculation characteristics of the lesions.

### Qualitative analysis of BI-RADS 4

All patients with BI-RADS grade 4 lesions were diagnosed by a senior breast radiologist with 15 years of experience. The senior radiologist relied on the morphology (size, margin and internal enhancement pattern) and time–signal intensity curve (TIC) to assess the lesions in isolation and was blinded to the pathological results. BI-RADS category 4 corresponds to a likelihood of malignancy between 2 and 95% and is further divided into categories 4a, 4b, and 4c. According to the American College of Radiology and BI-RADS in 2013, the definitions of 4a/4b/4c are as follows [7]: BI-RADS 4a indicates a low malignancy rate (2%–10% likelihood of malignancy), BI-RADS 4b indicates a moderate malignancy rate (10%–50% likelihood of malignancy), and BI-RADS 4c indicates a high malignancy rate (50%–95% likelihood of malignancy). BI-RADS 4a and 4b are classified as “benign”, and 4c is classified as “malignant”.

### Statistical analysis

All statistical analyses were carried out by using IBM SPSS Statistics software (version 19.0) and R software (version 3.5.1), with *P* < 0.05 indicating statistical significance. Student’s *t* test was used to evaluate the differences in pharmacokinetic parameters (within the Lesion area, the Peri area, and the BPE area) from high-/low-temporal-resolution DCE-MRI between the malignant and benign groups. Spearman’s correlation was used to analyze the relationship between pharmacokinetic parameters and high/low temporal resolution. The performance of the pharmacokinetic parameters in discriminating between benign and malignant lesions was evaluated by receiver operating characteristic (ROC) curve analysis. The AUC values were compared by the Delong test.

## Results

### Study population

There were 62 patients (average age, 51.82 ± 11.65 years) who underwent high-temporal-resolution DCE-MRI and 78 patients (average age, 47.29 ± 10.52 years) who underwent low-temporal-resolution DCE-MRI. Among these patients, fifty-six patients with BI-RADS 4 breast lesions (average age, 48.41 ± 11.73 years) were diagnosed and underwent high-temporal-resolution (37.5%, 21/56) or low-temporal-resolution (62.5%, 35/56) DCE-MRI. In BI-RADS 4 patients, the high-temporal-resolution DCE-MRI group included 9 (42.9%, 9/21) benign and 12 (57.1%, 12/21) malignant lesions; the low-temporal-resolution DCE-MRI group included 12 (34.3%, 12/35) benign and 23 (65.7%, 23/35) malignant lesions. The general characteristics of the patients are shown in Table 1.

**Table 1:** General characteristics of patients

Characteristics	H_DCE-MRI group	L_DCE-MRI group	*P*
Number	62	78	
Age (years)	51.82 ± 11.65	47.29 ± 10.52	0.025*
*Pathological type*			0.650
Invasive ductal carcinoma	33 (53.2%)	41 (52.6%)	
Ductal carcinoma in situ	6 (9.7%)	5 (6.4%)	
Fibroadenoma	13 (21.0%)	17 (21.8%)	
Intraductal papilloma	2 (3.2%)	7 (9.0%)	
Fibrocystic change	8 (12.9%)	8 (10.2%)	
*BI-RADS category*			0.166
BI-RADS 2	3 (4.8%)	0 (0.0%)	
BI-RADS 3	13 (21.0%)	20 (25.6%)	
BI-RADS 4a	6 (9.7%)	7 (9.0%)	
BI-RADS 4b	5 (8.1%)	16 (20.5%)	
BI-RADS 4c	10 (16.1%)	12 (15.4%)	
BI-RADS 5	21 (33.9%)	18 (23.1%)	
BI-RADS 6	4 (6.4%)	5 (6.4%)	

BI-RADS category diagnosed by the senior radiologist

*P < 0.05

### Pharmacokinetic parameters from DCE-MRI

**Fig. 3:**
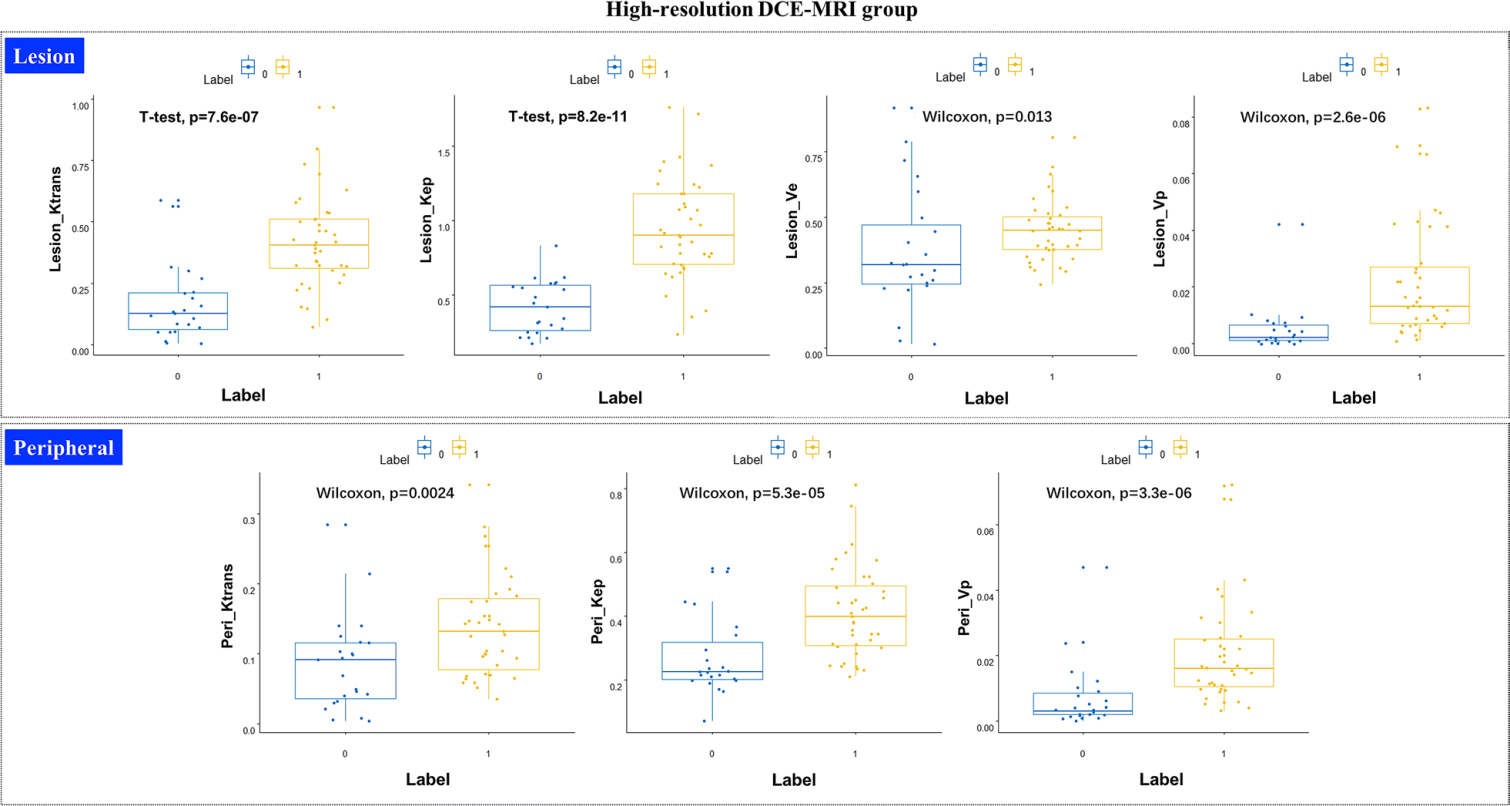
Boxplot of pharmacokinetic parameters between benign (Label 0) and malignant (Label 1) from high-temporal-resolution DCE-MRI

The pharmacokinetic parameters from high-temporal-resolution DCE-MRI were compared between benign and malignant lesions. The results showed that K^trans^, K_ep_, and V_p_ were significantly different in the intralesional (Lesion_K^trans^, Lesion_K_ep_, and Lesion_V_p_) and perilesional (Peri_K^trans^, Peri_K_ep_, and Peri_V_p_) areas (*P* = 0.000), but V_e_ was significantly different only in the intralesional (Lesion_V_e_) area (*P* = 0.013). There were no significant differences in the background parenchyma enhancement (BPE) area between the benign and malignant groups (*P* > 0.05; Table 2, Fig. 3).

**Table 2:** Pharmacokinetic parameters between benign and malignant lesions

Pharmacokinetic parameters	Lesion area			Peri area			BPE area		
	Benign	Malignant	*P*	Benign	Malignant	*P*	Benign	Malignant	*P*
*High-temporal-resolution DCE-MRI group*									
K^trans^	0.167 ± 0.156	0.416 ± 0.189	0.000*	0.085 ± 0.068	0.140 ± 0.073	0.002*	0.060 ± 0.070	0.044 ± 0.047	0.471
K_ep_	0.418 ± 0.177	0.943 ± 0.345	0.000*	0.271 ± 0.121	0.412 ± 0.143	0.000*	0.263 ± 0.192	0.254 ± 0.158	0.983
V_e_	0.371 ± 0.233	0.449 ± 0.119	0.013*	0.284 ± 0.154	0.335 ± 0.143	0.378	0.204 ± 0.123	0.189 ± 0.170	0.355
V_p_	0.005 ± 0.009	0.022 ± 0.020	0.000*	0.007 ± 0.010	0.020 ± 0.015	0.000*	0.003 ± 0.003	0.004 ± 0.006	0.988
*Low-temporal-resolution DCE-MRI group*									
K^trans^	2.311 ± 1.539	1.830 ± 1.185	0.141	1.916 ± 1.256	1.588 ± 0.858	0.452	2.764 ± 1.633	1.699 ± 1.141	0.005*
K_ep_	0.219 ± 0.203	0.415 ± 0.194	0.000*	0.235 ± 0.156	0.304 ± 0.169	0.081	0.304 ± 0.304	0.340 ± 0.198	0.164
Ve	0.454 ± 0.213	0.558 ± 0.188	0.052	0.309 ± 0.114	0.349 ± 0.117	0.120	0.585 ± 0.288	0.475 ± 0.177	0.059
V_p_	0.720 ± 0.280	0.642 ± 0.221	0.070	0.566 ± 0.217	0.403 ± 0.157	0.001*	0.663 ± 0.298	0.457 ± 0.196	0.002*

*BPE* background parenchyma enhancement, *Peri* perilesional

*P < 0.05

**Fig. 4:**
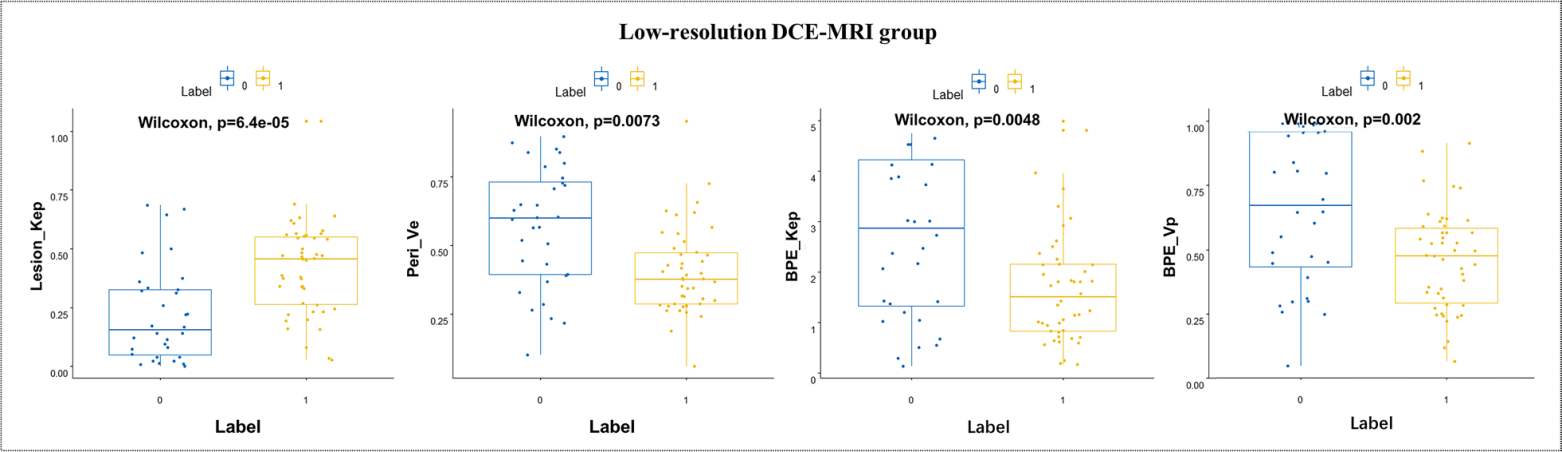
Boxplot of pharmacokinetic parameters between benign (Label 0) and malignant (Label 1) from low-temporal-resolution DCE-MRI

The pharmacokinetic parameters from low-temporal-resolution DCE-MRI were compared between benign and malignant lesions. The results showed that Lesion_K_ep_ in the intralesional area (*P* = 0.000), Peri_V_p_ in the perilesional area (*P* = 0.001), and BPE_K^trans^ and BPE_V_p_ in the BPE area (*P* = 0.005 and 0.002) were significantly different between lesion types (Table 2, Fig. 4).

### Association between pharmacokinetic parameters and high/low temporal resolution

**Fig. 5:**
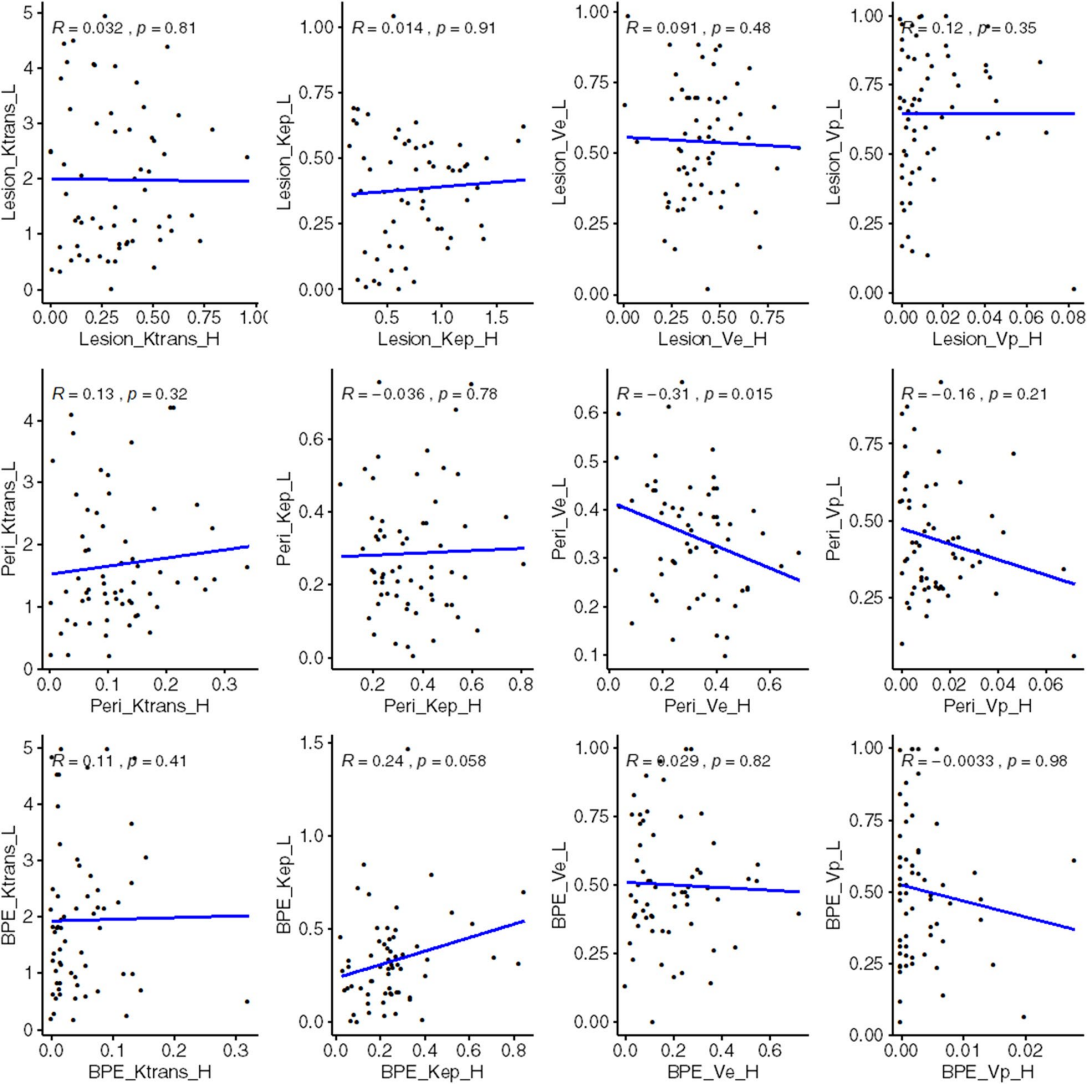
Association between pharmacokinetic parameters and high (H)/low (L)-temporal resolution

The associations between the same pharmacokinetic parameters on high- and low-temporal-resolution DCE-MRI were analyzed. The results showed that Peri_V_e_ was negatively associated with temporal resolution (r = -0.31, *P* = 0.015), while BPE_K_ep_ was positively associated with temporal resolution at the verge of significance (r = 0.24, *P* = 0.058). The other pharmacokinetic parameters showed no association with the temporal resolution of DCE-MRI (Fig. 5).

### Performance of pharmacokinetic parameters in discriminating between benign and malignant lesions

**Fig. 6:**
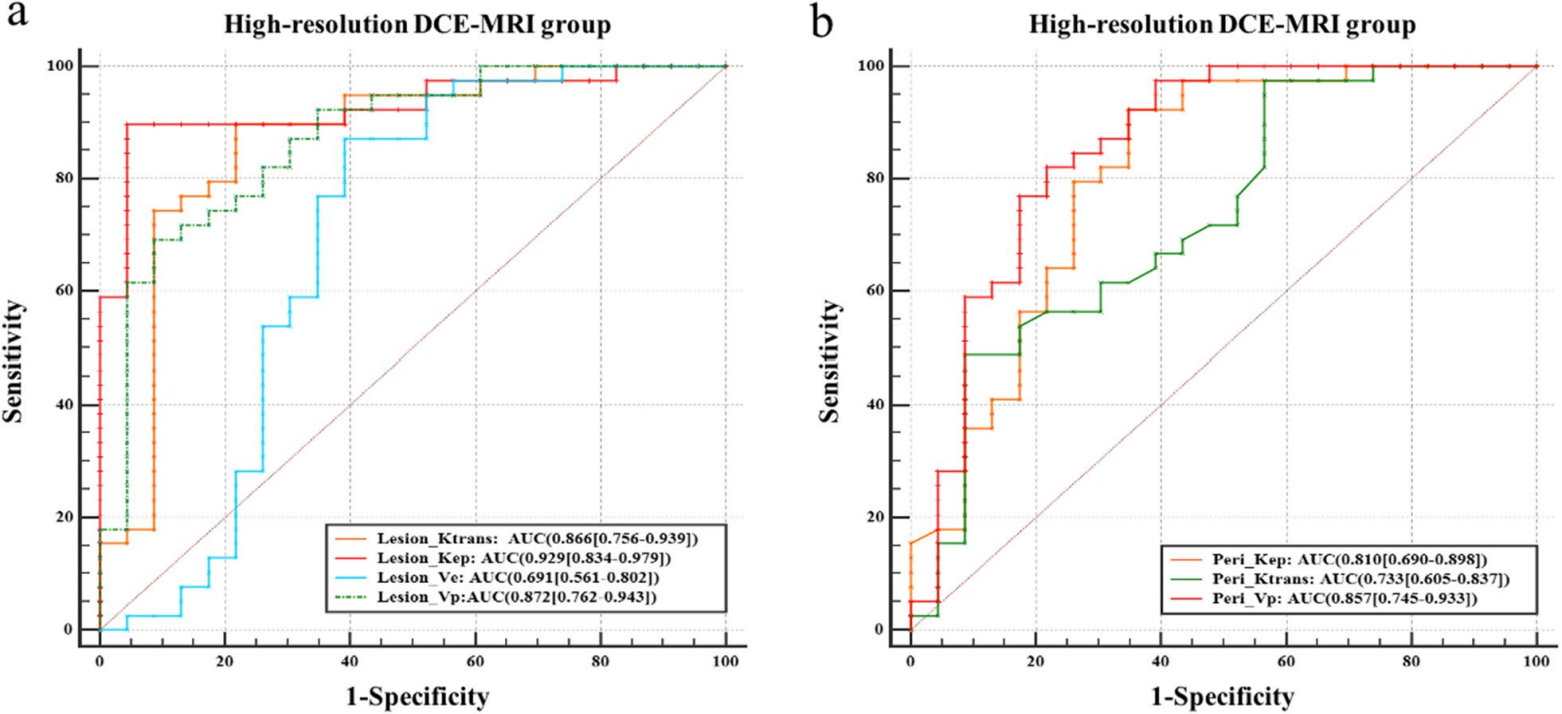
ROC curves of pharmacokinetic parameters in the intralesional area (**a**) and perilesional area (**b**) from high-temporal-resolution DCE-MRI

In the high-temporal-resolution DCE-MRI group, the four pharmacokinetic parameters (Lesion_K^trans^, Lesion_K_ep_, Lesion_V_e_ and Lesion_V_p_) in the intralesional area could differentiate benign and malignant breast lesions (AUC = 0.866, 0.929, 0.691 and 0.872); the AUCs of K^trans^, K_ep_, and V_p_ were all above 0.800, with Lesion_K^trans^ having the greatest AUC. Peri_K^trans^, Peri_K_ep_, and Peri_V_p_ in the perilesional area also provided good differentiation (AUC = 0.733, 0.810 and 0.857), with the AUC of Peri_V_p_ being the best. The results showed that the pharmacokinetic parameters of the BPE area had no ability to differentiate between benign and malignant lesions (Table 3, Fig. 6).

**Table 3:** Discriminative performance of pharmacokinetic parameters

	AUC	95% CI		SEN (%)	SPC (%)	Cutoff	Youden	*P*
*High-temporal-resolution DCE-MRI group*								
Lesion_K^trans^	0.866	0.756–0.939		89.7	78.3	> 0.214	0.680	< 0.0001
Lesion_K_ep_	0.929	0.834–0.979		89.7	95.7	> 0.618	0.854	< 0.0001
Lesion_V_e_	0.691	0.561–0.802		87.2	60.8	> 0.325	0.480	0.025
Lesion_V_p_	0.872	0.762–0.943		69.2	91.3	> 0.009	0.605	< 0.0001
Peri_K^trans^	0.733	0.605–0.837		97.4	43.5	> 0.049	0.409	0.0007
Peri_K_ep_	0.810	0.690–0.898		92.3	65.2	> 0.240	0.575	< 0.0001
Peri_V_p_	0.857	0.745–0.933		82.1	78.3	> 0.008	0.603	< 0.0001
*Low-temporal-resolution DCE-MRI group*								
Lesion_K_ep_	0.767	0.658–0.855		69.6	78.1	> 0.334	0.477	< 0.0001
Peri_V_p_	0.726	0.613–0.821		84.8	59.4	≤ 0.548	0.442	0.0004
BPE_K^trans^	0.687	0.572–0.787		80.4	59.4	≤ 2.369	0.398	0.005
BPE_V_p_	0.707	0.593–0.805		89.1	56.2	≤ 0.639	0.454	0.0014

*AUC* area under the curve, *BPE* background parenchyma enhancement, *CI* confidence interval, *Peri* perilesional, *SEN* sensitivity, *SPC *specificity

**Fig. 7:**
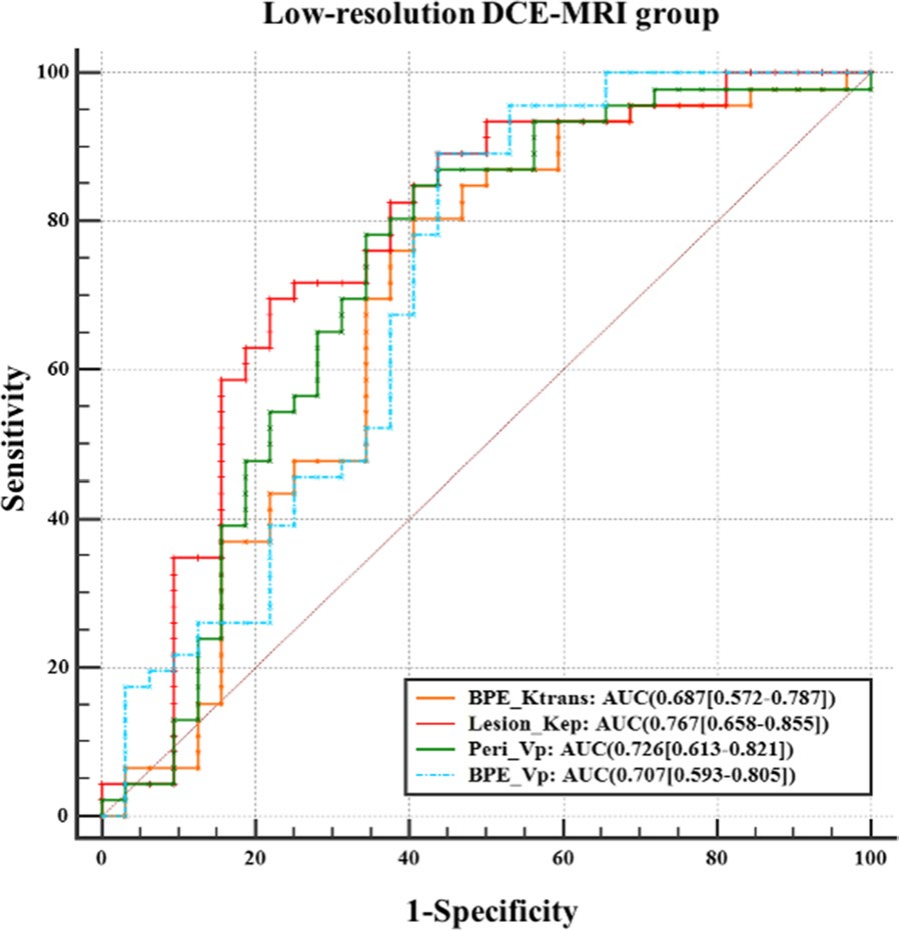
ROC curves of pharmacokinetic parameters in the intralesional, perilesional, and BPE areas from low-temporal-resolution DCE-MRI

In the low-temporal-resolution DCE-MRI group, Lesion_K^trans^ did not have discriminative ability, but Lesion_K_ep_ could differentiate between benign and malignant breast lesions with low sensitivity (AUC = 0.767, SEN = 69.6%, SPC = 78.1%). Peri_V_p_ and BPE_V_p_ achieved differentiation with low specificity (AUC = 0.726 and 0.707, SEN = 84.8% and 89.1%, SPC = 59. 4% and 56.2%, respectively). BPE_K^trans^ also provided differentiation with low specificity (AUC = 0.687, SEN = 0.804, SPC = 0.594) (Table 3, Fig. 7).

### Comparison of diagnostic performance between pharmacokinetic parameters and a radiologist in patients with BI-RADS 4

**Fig. 8:**
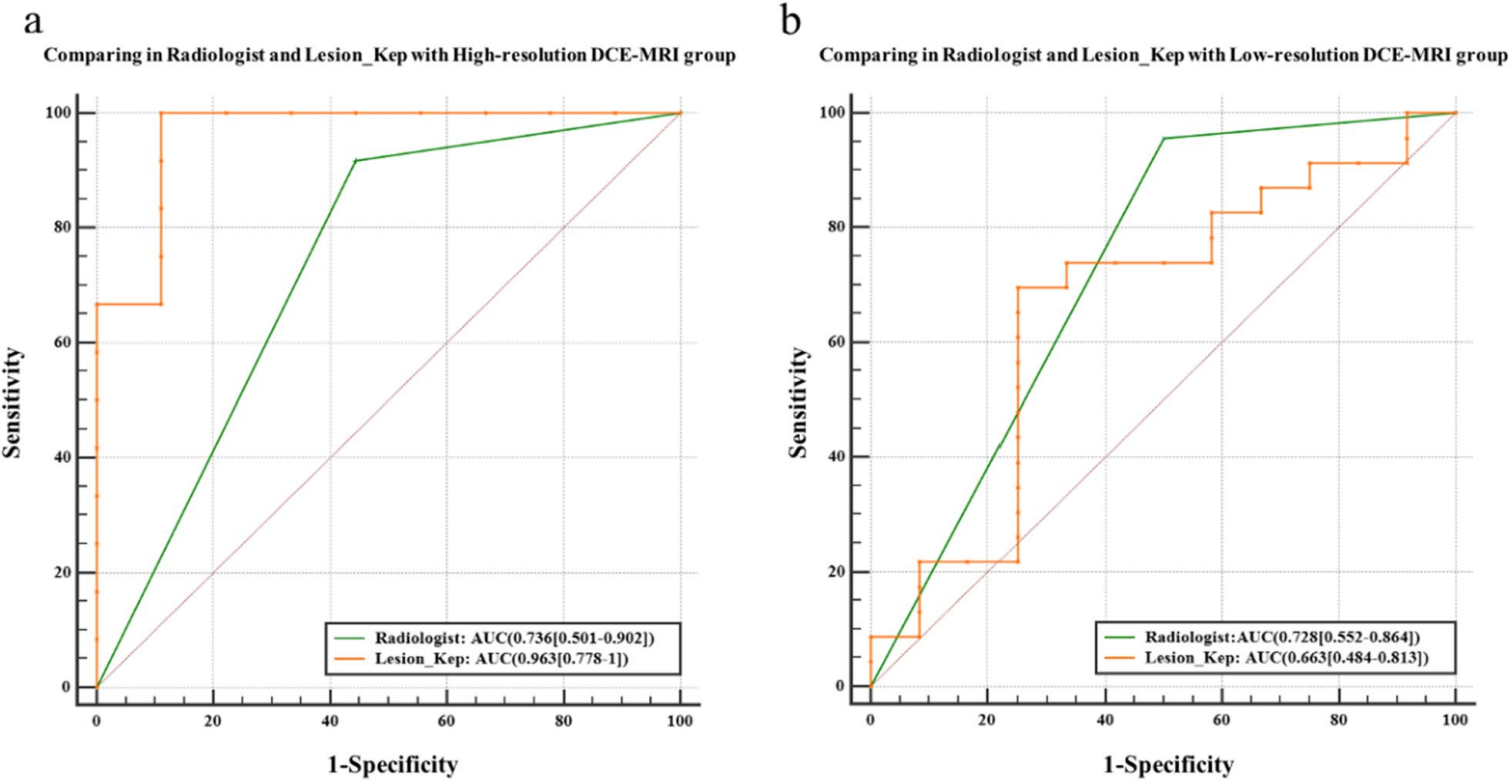
ROC curves of a pharmacokinetic parameter from high (**a**) and low-temporal-resolution (**b**) DCE-MRI and radiologist in BI-RADS 4 lesions

For 56 patients with BI-RADS 4 breast lesions, the optimal quantitative pharmacokinetic parameters (Lesion_K_ep_) in the high- and low-temporal-resolution DCE-MRI group were compared with those of the senior radiologist. In the high-temporal-resolution group, the results showed that the AUC values of Lesion_K_ep_ and the radiologist were 0.963 (95% CI 0.778–1.00) and 0.736 (95% CI 0.501–0.902), respectively, and the sensitivity and specificity of Lesion_K_ep_ were high. The DeLong test was conducted, and the results showed that there was a significant difference between the two curves (*P* = 0.04; Table 4**, **Fig. 8a**)**.

**Table 4:** The AUCs of pharmacokinetic parameters and a radiologist in BI-RADS 4 lesions

	AUC	95% CI	SEN (%)	SPC (%)	*P*^#^
*High-temporal-resolution DCE-MRI group (n = 21)*					
Radiologist	0.736	0.501–0.902	91.7	55.6	0.04*
High_Lesion_K_ep_	0.963	0.778–1.00	100.0	88.9	
*Low-temporal-resolution DCE-MRI group (n = 35)*					
Radiologist	0.728	0.552–0.864	95.7	50.0	> 0.05
Low_Lesion_K_ep_	0.663	0.484–0.813	69.6	75.0	

*AUC* area under the curve, *CI* confidence interval, *SEN* sensitivity, *SPC* specificity

^#^*P* value was Delong test result by comparing AUC value

*P < 0.05

In the low-temporal-resolution DCE-MRI group, the results showed that the AUC values of Lesion_K_ep_ and the senior radiologist were 0.663 (95% CI 0.474–0.813) and 0.728 (95% CI 0.552–0.864), respectively. The DeLong test was conducted, and the results showed no significant difference between the two curves (*P*> 0.05). There was also no significant difference in the senior radiologist's diagnostic performance between the high- and low-temporal-resolution groups (*P*> 0.05). The diagnostic efficacy of low-temporal-resolution Lesion_K_ep_ was lower than that of the radiologist, but its specificity was higher (Table 4, Fig. 8b).

## Discussion

In this study, we found that high-temporal-resolution DCE-MRI was superior to conventional low-temporal-resolution DCE-MRI in diagnostic performance for BI-RADS category 4 breast lesions based on pharmacokinetic parameters from intralesional and perilesional areas, but not parameters from background parenchymal enhancement. Low-temporal resolution DCE-MRI, which is merely similar to perfusion imaging, is not suitable for perfusion analysis and cannot truly reflect the microvascular state of lesions. Moreover, Peri_V_e_ and BPE_K_ep_ had a significant correlation between high and low temporal resolutions.

Regarding hemodynamic parameters from high-temporal-resolution DCE-MRI, our results showed that some parameters had good discrimination performance in the intralesional area (AUC_K^trans^, K_ep_, and V_p_ = 0.866, 0.929, and 0.872, respectively) and perilesional area (AUC_K^trans^, K_ep_, and V_p_ = 0.733, 0.810, and 0.857) but no differences in the BPE region. Among the various parameters, K^trans^ reflects the movement of contrast agent from the plasma to the EES and depends on the vascular permeability and vascular density of the lesion; K_ep_ is the rate constant of movement from the EES to the plasma and depends on vascular permeability; and V_p_, the fractional plasma volume, depends on vascularity [16, 17]. In our study, the results showed that K_ep_ and V_p_ in the lesion and perilesional area had especially stable performance (AUC > 0.8), which was slightly different from the findings of some studies [18, 19]. Clinically, K^trans^ might better represent the actual vascular features of a tumor. However, some studies have shown that K_ep_ can better reflect the difference between benign and malignant lesions [20–22], while V_p_ can reflect lesion vascularity [17]. We believe that the malignant lesions had more angiogenesis than the benign lesions and that the area surrounding the malignant lesions had higher vessel wall permeability, resulting in the differences in K^trans^, K_ep_ and V_p_ between benign and malignant lesions [9]. Meanwhile, our results also showed that the diagnostic performance of K_ep_ was better than that of K^trans^. The reason may be that K^trans^ is more likely to be influenced by abnormal blood perfusion, while K_ep_ is not [23]. Thus, we believe that K^trans^, K_ep_ and V_p_ obtained from high-temporal-resolution DCE-MRI may provide the most accurate indication of tumor vascularity and capillary permeability to distinguish benign from malignant lesions.

Relative to high-temporal-resolution DCE-MRI, some parameters (Lesion_K_ep_, Peri_V_p_, BPE_K^trans^ and BPE_V_p_) from low-temporal-resolution DCE-MRI also had differentiation ability (AUC < 0.8), but at a lower level of performance. This result indicated that high-temporal-resolution DCE-MRI may be more useful to doctors than low-temporal-resolution DCE-MRI in improving the assessment of BI-RADS 4 breast lesions; in this respect, our findings are in line with those of other studies [11, 20–22, 24, 25]. When the temporal resolution of DCE-MRI improved, the diagnostic performance of quantitative parameters improved as well, especially between 15 and 60 s; our results were similar [24]. Meanwhile, there were significant differences in tumor-associated interstitial flow velocity, blood pressure, and vascular extraction rate between malignant and benign lesions [26, 27]. Thus, some pharmacokinetic parameters obtained from high-temporal-resolution DCE-MRI could be better at differentiating malignant and benign breast lesions than those obtained from low-temporal-resolution DCE-MRI.

Based on the AUC values of pharmacokinetic parameters from high- and low-temporal-resolution DCE-MRI, our results showed that the Lesion_K_ep_ parameter obtained from DCE-MRI had the best diagnostic performance. Regarding clinical practice, we found that the radiologist had high sensitivity and low specificity compared to Lesion_K_ep_, indicating that a radiologist is likely to overdiagnose the disease. The Lesion_K_ep_ from low-temporal-resolution DCE-MRI offered similar diagnostic efficacy to the senior radiologist and had higher specificity. The results also suggested that low-temporal-resolution DCE-MRI was more likely than a radiologist to miss the diagnosis. Importantly, the Lesion_K_ep_ from high-temporal-resolution DCE-MRI markedly outperformed the senior radiologist and had higher sensitivity and specificity. Therefore, the quantitative parameters were more effective than subjective diagnoses, and the K_ep_ of the lesion area obtained from high-temporal-resolution DCE-MRI may be more suitable for the assessment of BI-RADS 4 breast lesions.

Interestingly, the hemodynamic parameters of the BPE area from high-temporal-resolution resolution DCE-MRI were not effective for discrimination, but the K^trans^ and V_p_ of the BPE area from low-temporal-resolution DCE-MRI were (AUC < 0.8). BPE is associated with the microvascular density and glandular ratio of fibroglandular tissue, which could explain the positive association with neoplasia [12, 14, 28]. Some studies have found that higher levels in the BPE area are associated with higher rates of abnormal findings (BI-RADS category 0/3/4/5) [28]. However, BPE also has a dynamic presentation, and its distribution in a woman’s breast tissue is sensitive to lactation and the phases of the menstrual cycle [28, 29]. Kim et al. [30] found that K^trans^ (AUC = 0.687) and V_p_ (AUC = 0.648) in the BPE area could help to differentiate malignant lesions from benign lesions. However, high-temporal-resolution DCE-MRI (a temporal resolution of 11 s) did not show diagnostic performance based on the BPE area. Therefore, we speculate that the differences in K^trans^ and V_p_ in the BPE area from low-temporal-resolution DCE-MRI were due to hormonal effects or the limited sample size of this study. BPE may not be useful for discriminating between benign and malignant lesions; more patients are needed to clarify this point in the future.

On the other hand, our study showed that Peri_V_e_ was negatively associated with temporal resolution, while BPE_K_ep_ had a borderline-significant positive association with temporal resolution. Nonetheless, these two parameters showed no ability to discriminate between benign and malignant lesions. Ya et al. [31] showed that V_e_ values showed a progressive increase with the progressive filling of the EES by contrast agent passing through vessel walls over time. Moreover, Kuhl et al. [32] found that the values of V_e_ could be influenced by the extracellular space and disorganized microarchitecture. Nevertheless, Matsukuma et al. [33] found no significant differences in K^trans^, V_e_, or K_ep_ with different temporal resolutions. In our study, the correlation coefficients of Peri_V_e_ (r = -0.31) and BPE_K_ep_ (r = 0.24) were smaller than 0.5, indicating that the relationship was weak. This may be related to the number of patients. Therefore, we believe that the correlation between hemodynamic parameters and different temporal resolutions needs to be further investigated.

There were several limitations in this study. First, the sample size was relatively small, and further study with a larger number of patients is needed to confirm our results. Second, high-temporal-resolution DCE-MRI scans still need further optimization of scanning time and have the same slice thickness as conventional DCE-MRI. Third, the current study did not analyze qualitative and semiquantitative factors, such as time–intensity curves, washin/washout rate, and signal enhancement ratio. In our subsequent work, we will perform a prospective study to compare the diagnostic performance of qualitative analysis and semiquantitative analysis in conventional low-temporal-resolution DCE-MRI. Fourth, the AIF is essential to calculate kinetic parameters; therefore, we must further compare the different methods for drawing this function from conventional low-temporal-resolution DCE-MRI, such as drawing the AIF from the thoracic aorta or basing it on a reference tissue.

## Conclusions

In summary, high-temporal-resolution DCE-MRI was superior to conventional low-temporal-resolution DCE-MRI in the differential diagnosis of BI-RADS category 4 breast lesions to avoid unnecessary biopsy; several pharmacokinetic parameters (K^trans^, K_ep_, V_p_) were increased in the internal and peripheral areas of malignant lesions on high-temporal-resolution DCE-MRI. These parameters, especially the intralesional K_ep_ parameter, were useful for differentiating between benign and malignant lesions. Low-temporal-resolution DCE-MRI, which is merely similar to perfusion imaging, is not suitable for perfusion analysis and cannot truly reflect the microvascular state of lesions.

## Data Availability

The datasets used and/or analyzed during the current study are available from the corresponding author on reasonable request.

## Abbreviations

AUC: Area under curve
BPE: Background parenchymal enhancement
DCE-MRI: Dynamic contrast-enhanced magnetic resonance imaging
EES: Extravascular extracellular space
K^trans^: Volume transfer constant
K_ep_: Reverse reflux rate constant
ROC: Receiver operating characteristic
ROI: Region of interest
V_e_: Fractional EES volume
V_p_: Fractional plasma volume
